# A Treatise on Headache and Neuralgia

**Published:** 1891-01

**Authors:** 


### BOOK NOTICES.

A Treatise on Headache and Neuralgia, including Spinal Irritation and a Disquisition on Normal and Morbid Sleep. By J. Leonard Corning, M. A., M. D. With an Appendix on Eye Strain, a Cause of Headache. By David Webster, M. D. Illustrated. Second edition. New York: E. B. Treat, 5 Cooper Union. London: H. K. Lewis, 136 Gower St., 1890. Price, $2.75.

From this volume we may get some practical facts and many theories regarding the various subjects treated. As Hahnemannians we should have the desire to know what is thought by the best minds of the old school, particularly on the subjects treated in this work. The author, Dr. Corning, is a leading New York practitioner, and we notice that his work is considered of the best by his fellow-allopaths. In this work are given the various kinds of headache, the intracranial forms being treated in the first part, while the second treats of neuralgia. When we come to the next division, the treatment, we can only pity the writer for knowing nothing of Hahnemannian Homœopathy; but to his patients we give more sympathy. The chapter on Spinal Irritation is worth, in our estimation, more than any other portion, for, as we have said in another place, this is a subject of moment to every physician. Dr. Webster's short chapter on "Eye Strain" is a practical one, giving illustrative cases. We should all know that many neuralgic symptoms and headaches are due to abnormal refraction, and properly adjusted glasses only can give relief in such cases. Hence, where our remedies fail to give permanent relief, such a condition should be suspected. In this work may be seen just what symptoms may be due to this cause, and what results from their proper treatment. At the same time, we should bear in mind that many cases that seemingly need glasses can only be made well by the properly selected homœopathic remedy. We have frequently seen apparent high degrees of hypermetropia and myopia disappear by the use of the indicated medicinal remedy alone. Therefore, we should never fail to give our patients the benefit arising from the consideration of all their symptoms.

